# Sex-specific relationship patterns between body morphology and maturity status with change of direction and agility in elite adolescent volleyball players

**DOI:** 10.1038/s41598-024-64190-6

**Published:** 2024-06-07

**Authors:** Dawid Koźlenia, Marek Popowczak, Pavol Horička, Jaromir Šimonek, Jarosław Domaradzki

**Affiliations:** 1 Faculty of Physical Education and Sport, Wroclaw University of Health and Sport Sciences, I.J. Paderewskiego 35, 51-612 Wroclaw, Poland; 2 Department of Physical Education and Sport, Constantine the Philosopher University in Nitra, Tr. A. Hlinku 1, 94901 Nitra, Slovakia

**Keywords:** Ageing, Musculoskeletal system

## Abstract

This study explored sex-specific patterns in the relationship between maturity, body morphology, and change of direction (COD) and agility (AG) in adolescent volleyball players. The sample comprised 22 males and 24 females aged 15–17 with at least 4 years of sports experience. Measurements included body height, weight, muscle and fat mass, leg length, and center of mass. The study determined the Age of Peak Height Velocity (APHV) and evaluated sensorimotor ability through pre-planned COD and not-planned AG five-time shuttle runs, measuring the time difference as the index of reactivity. Positive correlations were found between COD and AG with fat mass index (FMI) in boys, while negative relative lower limb length (rLL) correlated with COD. In females, age of peak height velocity (APHV) showed a significant negative relationship with sensorimotor ability. Linear and polynomial regressions confirmed predictive and curvilinear relationships, respectively. Cluster analysis identified different associations in boys and girls, emphasizing sex-specific patterns. Body fat percentage had a negative impact on COD-AG in boys, while the optimal lower limb length proportion positively influenced COD due to enhanced maneuverability. Maturation affected sensorimotor abilities in girls. The findings suggest a need for a tailored approach to COD-AG development based on sex-specific considerations in adolescent volleyball players.

## Introduction

Agility (AG) is a swift, whole-body movements involving shifts in speed or direction prompted by a stimulus^1^. Change of direction (COD) is considered a planned and closed skill, does not necessitate a response to a stimulus, and should be distinguished and studied separately from AG^2,3^. AG is a complex motor ability, which fundaments are still explored^4^. Many results refer only to simple associations. A more complex view considering the form and multivariate relationship may deepen the view of agility to better understand this motor ability for its development^5^. The difference between performing the same task in a planned and non-planned manner is referred to as the index of reactivity. This index indicates sensorimotor ability, which allows for behavior modification in response to evolving environmental cues, ensuring the preservation of suitable goal-oriented motor skills^6^. For the effective development of COD-AG, a comprehensive understanding of the associations, forms, and structures is essential, particularly concerning sex-specific conditions during adolescence, which is a time of comprehensive and rapid changes in body structure and function^7^.

These skills are crucial in team sports such as volleyball, and their development states a base to maximize performance^8^. Volleyball requires continuous awareness of reactions in still-changing environments as well as quick movement in the planned tasks associated with tactically performed actions^9^. Knowing the factors affecting the COD-AG development process, especially in adolescent groups during physical, mental, and sports development, is crucial^10^.

Anthropometric factors, such as body dimensions and morphology, influence AG and COD^11–14^. These outcomes are particularly affected by maturity, characterized by rapid changes in body morphology, considering sex-specific conditions^7^. The potential relationships mentioned above may be significantly different based on visible differences between males and females. Especially when considering adolescents throughout their maturation status^15–17^. Age of peak height velocity (APHV) is a maturity indicator^15^. There is increasing application in studies focusing on sports, performance, and physical activity among youth from late childhood through adolescence. This approach emphasizes the need to consider individual differences in biological maturation^16^.

Many studies have considered typical factors such as body height, weight, or its ratio as COD-AG determinants, but results are not univocal^12,13^. The form of these associations may not be linear, so looking for optimal values is needed^5^. However, it would also be valuable to investigate more specific factors such as limb length (LL), the center of mass (COM), body fat, and muscle mass concerning sex-specific conditions, which would provide deeper insight into AG-COD fundaments^18^. Theoretical considerations suggest that body morphology and segment lengths might significantly impact COD and AG performance^19^. Studies investigating agility (AG) and change of direction (COD) have observed a consistent trend, indicating that individuals who perform well on COD or AG tests tend to exhibit lower body mass^20^. While the significance of body fat and muscle mass adeptness in rapid directional changes seems apparent, the precise relationship between these variables remains uncertain^21^. Shorter LL is a potential factor that could influence multidirectional locomotion tasks^22^. Some research indicates a connection between limb length and specific sporting movements, such as lunges typical in directional movement changes^23^. LL, a pivotal factor affecting step length, might improve sprint performance by enabling longer strides during sprinting^24^. However, it remains unclear how this feature affects COD and AG^25^.

Lower body height in shorter players and a lower COM tend to correlate with better results in CODS and agility assessments^26^. Theoretically, an individual with a lower center of gravity might apply horizontal force more rapidly than a taller athlete. The shorter individual would require less time to lower their center of gravity before executing a lateral direction change, potentially resulting in quicker direction changes. Exploring this possibility through quantitative biomechanical measurements could yield valuable insights^1,2,18^.

In the physical development of adolescents, maturation and the morphological features of their bodies hold considerable importance. Especially in team sports such as volleyball, locomotion based on fast movement is crucial during the game and development during youth training^27^. However, there’s a lack of comprehensive studies exploring the form and structure of connections between maturation, body dimension, morphology, and COD-AG tasks in sex-specific terms in volleyball players. Therefore, this study aimed to explore COD and AG sex-specific relationship patterns with maturity and body morphology. More precisely, we want to investigate (1) What are simple associations between factors? (2) What functional form do these relationships take? (3) What pattern of associations are presented by males and females? This study provides deeper insight into the basis of COD and AG development in adolescent volleyball players considering sex. The obtained results show which parameters are critical for COD-AG skills, what has meaning in sport selection, which parameters limit or are more favorable to develop performance, and what allows the introduction of related training resources.

## Material and methods

### Ethical approval

Ethical approval for this study was granted by the Research Bioethics Committee of the Faculty Senate of the Wroclaw University of Health and Sport Sciences (number 06/2018) and adhered to the ethical principles outlined in the Declaration of Helsinki. Before the study, all participants and their legal guardians provided written informed consent after thoroughly explaining the research’s purpose and characteristics.

### Study design

This study has a cross-sectional design.

### Participants

The study sample was non-probabilistic and recruited based on clearly described criteria. Measurements were conducted over the following two weeks. In the follow-up, 49 players were identified, but due to previous injury, 3 subjects were excluded (2 boys and 1 girl). Therefore, the study encompassed 46 elite adolescent volleyball athletes, 24 females and 22 males. The inclusion criteria were: (1) calendar age (15–17), (2) sports age (minimum four years), (3) lack of physical injury minimum 28 days before measurements. The individuals performed specific sports training 4–6 days (90–120 min) per week with one match. The detailed characteristics of study participants are in Table 1 in the results sections. All participants were volunteers.

**Table 1:** Detailed characteristics of study sample. Student T-test results for independent samples. *Statistically different values *p* < 0.05.

Variable	Men	Women	Statistics		
	Mean ± SD (95% CI)	Mean ± SD (95% CI)	t	p	ES
Calendar age (years)	16.85 ± 0.55 (16.61–17.09)	16.67 ± 1.31 (16.12–17.22)	− 0.61	0.54	0.2
Sport age (years)	6.55 ± 1.53 (5.87–7.23)	6.92 ± 2.08 (6.04–7.8)	0.68	0.50	0.2
Maturity Offset	3.28 ± 0.53 (3.05–3.52)	4.57 ± 1.04 (4.13–5.01)	5.23	< 0.01*	1.2
Age of Peak Height Velocity	13.12 ± 0.45 (12.93–13.32)	11.55 ± 0.58 (11.31–11.8)	− 10.26	< 0.01*	1.6
Centre of Mass (cm)	103.27 ± 3.38 (101.77–104.7)	96.67 ± 2.85 (95.46–97.87)	− 7.18	< 0.01*	1.4
relative lower limb length (cm)	0.48 ± 0.01 (0.47–0.48)	0.47 ± 0.01 (0.47–0.48)	− 1.63	0.19	0.5
Body Height (m)	1.85 ± 0.06 (1.83–1.88)	1.74 ± 0.05 (1.72–1.77)	− 6.77	< 0.01*	1.4
Body Weight (kg)	75.38 ± 7.84 (71.9–78.86)	67.35 ± 11.03 (62.69–72.01)	− 2.82	0.07*	0.8
Bmi (kg/m^2^)	21.96 ± 2.32 (20.93–22.99)	22.18 ± 4.00 (20.49–23.88)	0.23	0.82	0.06
FMI (body fat kg/m^2^)	2.42 ± 1.49 (1.76–3.08)	4.89 ± 2.83 (3.69–6.08)	3.65	0.01*	0.9
SMI (body fat kg/m^2^)	11.16 ± 0.87 (10.77–11.55)	9.65 ± 0.93 (9.25–10.04)	− 5.69	< 0.01*	1.3
Change of Direction (s)	14.03 ± 0.71 (13.72–14.35)	15.695 ± 0.721 (15.391–15.999)	7.87	< 0.01*	1.5
Reactive Agility (s)	17.79 ± 1.1 (17.3–18.27)	19.129 ± 0.775 (18.802–19.456)	4.83	< 0.01*	1.6
Index of reactivity (s)	3.75 ± 0.73 (3.43–4.08)	3.434 ± 0.769 (3.11–3.759)	− 1.44	0.11	0.5

### Measurements

The anthropometry measures were taken according to the International Society for the Advancement of Kinanthropometry (ISAK), which clearly described the procedures required to perform anthropometry measures^28^. Each anthropometric measurement was conducted privately in a separate room by an experienced specialist.

### Body morphology

Body morphology measurements were conducted a day before the assessment of high-speed abilities and scheduled for the morning. Body height in standing and sitting positions was measured with an accuracy of 0.1 cm using a Swiss anthropometer (GPM Anthropological Instruments). The lower limb measurements were performed based on differences in total body height and sitting height, and the relative ratio between total body height and lower limb longevity was calculated.

- Relative leg length (rLL) = (stature-sitting height)/stature

A bioelectrical impedance analysis (BIA) was performed to analyze body mass and composition. Due to its confirmed high reliability, the InBody230 device (InBody Co., Ltd., Cerritos, CA, USA) was utilized for this purpose^29^. This device provides data on total body mass (in kilograms), body fat percentage, and muscle mass (in kilograms). To ensure consistency in bio-impedance measurements, protocols outlined by^30^ were strictly followed. Participants were instructed to maintain specific conditions, including abstaining from physical activities 24 h before the tests, fasting for at least eight hours, limiting fluid intake three hours before measurements, and emptying their bladder immediately before assessment. From the acquired data, various indices were computed using equations:

- Body Mass Index (BMI) = body mass (kg)/body height^2^ (m^2^)
- Fat Mass Index (FMI) = body fat mass (kg)/body height^2^ (m^2^)
- Skeletal Muscle Mass Index (SMI) = body skeletal muscle mass (kg)/body height^2^ (m^2^)

### Age at peak height velocity (APHV)

The assessment of the pace of biological growth, indicating the moment of maturity for both sexes in EG and CG, utilized formulas Moore et al.^31^ developed to predict Age at Peak Height Velocity (APHV). These predictions were made through sex-specific regression equations:

- For girls: Maturity offset (MO) = − 7.709133 + (0.0042232 * (age * BH)).
- For boys: MO = -7.999994 + (0.0036124 * (age * BH)).

Age at Peak Height Velocity (APHV) calculation involved subtracting the maturity offset (MO) from the individual’s calendar age for all participants.

### The center of mass (COM)

**Figure 1:**
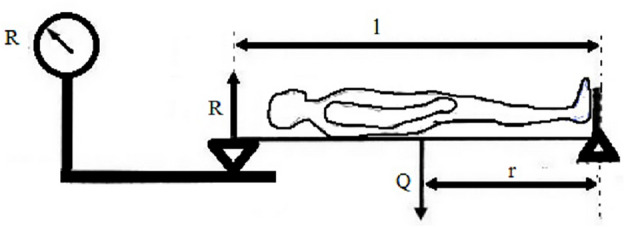
The center of body mass (COM) estimation uses a one-sided lever.

The center of mass (COM) was calculated with the body using the method of a one-sided lever described by Bober and Zawadzki et al.^32^. A lever is a rigid beam supported at one point, allowing it to perform rotational movements freely. Moments of forces acting on the lever induce rotational motion. The lever is in equilibrium if these moments balance each other (meaning their sum is zero). This property of a lever can be utilized to determine the position of the center of gravity of objects placed on it. A person applied to the lever at a distance from the pivot point generates a force moment. This moment causes the lever to rotate clockwise. However, to maintain equilibrium, it is necessary to counteract the effect by applying an opposing moment of equal magnitude. This balancing allows for the determination of the overall center of gravity of the human body using the one-sided lever method using the equation: r(COM) = (R*l)/Q whereas r—center of mass [cm]; l—leveler longevity [cm]; R—weight indication [kg]; Q—total body mass [kg]. Figure 1 presents the testing, and an appropriate equation was provided.

### Change of direction (COD) and Agility (AG)

**Figure 2:**
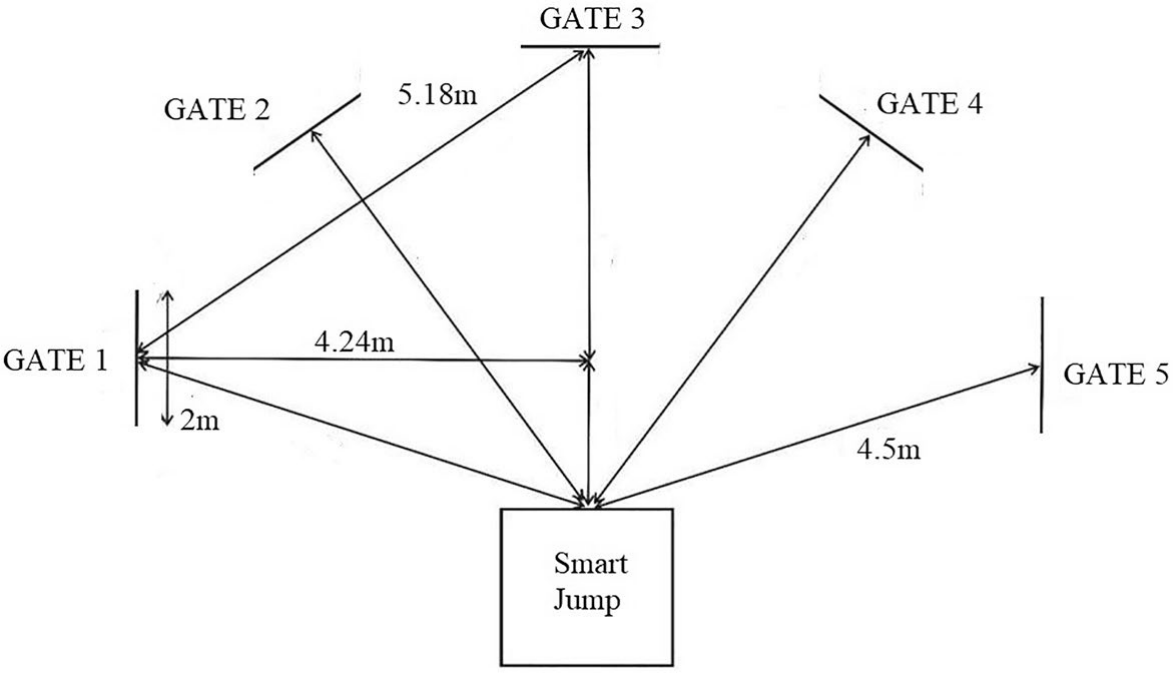
Five-time shuttle run test scheme.

Before the measurements commenced, participants underwent a standardized 15-min warm-up routine. Subsequently, the ‘five-time shuttle run to gates’ test was performed to determine Change of Directions (CODs) and Reaction Ability (RA). This test utilized a Fusion Smart Speed System (Fusion Sport, Coopers Plains, QLD, Australia), featuring five gates equipped with photocells and an infrared transmitter, a Smart Jump mat integrated with photocells and a radio frequency identification (RFID) reader, along with dedicated computer software (Fig. 2). The testing apparatus accurately measured running time to 0.001 s, with data recorded on a computer PDA (HP iPAQ 112) linked to the participants’ names. The ‘five-time shuttle run to gates’ test assessed running time involving directional changes triggered by a light stimulus using the ‘stop–go’ method. This system, as proposed by Popowczak et al.^33^, activated lights in fixed (pre-planned) or randomly chosen gates (non-planned). Participants completed five runs between the photocells and reflectors and returned to the starting mat. Photocell-integrated mats were positioned at the start and finish lines (Fig. 2). Upon both feet touching the central mat area, a light signal immediately directed participants to the designated gate. In the pre-planned ‘five-time shuttle run to gates’ test, assessing CODs speed, participants ran to gates in a predetermined sequence (from 1 to 5). The COD angle within the gate was approximately 180°, while the maneuver on the mat jump was executed at an angle of around 135°. This test was repeated twice, and the best overall duration was utilized for analysis. In contrast, the non-planned ‘five-time shuttle run to gates’ test, measuring RA, directed participants to randomly selected gates. Each participant had a unique gate sequence, but the distances remained consistent. During AG sprints, participants were instructed to avoid predicting the exit gate. This test was repeated twice, and the best overall duration was considered for analysis. All assessments occurred within a sports hall without specifying player positions in the subsequent analysis. The time difference between tests provides an index of reactivity^6^, indicating sensorimotor ability.

### Statistics

The data normality distribution was checked by Shapiro- Wilk Test. The means, standard deviations (SD), and confidence intervals (95%CI) were calculated for the analyzed variables. The t-test comparison was conducted between the sexes. The Cohen’s d statistic was calculated to assess the effect size. The interpretation was based on Cohen’s suggestion and stated as small effect size (d = 0.2), medium effect size (d = 0.5), and large effect size (d = 0.8)^34^. Pearson correlation was calculated to investigate the simple relationship between analyzed parameters. Regression models were employed to explore the nature of the relationships between the analyzed parameters. Simple linear regression and binomial curvilinear regression were constructed. In each model, coefficients (x), squared coefficients (x^2^) in the regression function, R-squared (R^2^) as the coefficient of determination, and p-values were computed to assess the goodness-of-fit of the model. Based on the equation y = ax^2^ + bx + c, the following formula was used to calculate the peak value: max = − b/2a. In the last step, cluster analysis was employed to group variables with similar characteristics within clusters and distinguish those with dissimilar features outside the clusters. This is a multidimensional technique for assessing the interconnections among variables. Typically, cluster analysis involves a uniform set of variables (measured on the same scale, such as continuous variables). In such cases, a straightforward standardization method is typically satisfactory for meeting the assumptions necessary for cluster analysis. Ward’s method was utilized for grouping, generating clusters with minimal variance within and maximum variation between them. The Euclidean distance was used. The resulting dendrogram illustrates groups of variables with the least differences connected at a specific level of similarity, revealing the internal structure of associations between measured features. The significance level was set at *p* < 0.05. Statistica v13.3 (Statsoft Polska, Cracow, Poland) was used for statistical analyses.

## Results

Table 1 presents maturity and body morphology measurements and COD-AG results for males and females. The detailed comparison revealed that girls are more mature. However, significant sex differences in body morphology were observed. Boys were characterized by higher body dimensions and lower body fat than girls, with better times in motor tests, but the reactivity index tended to be better in girls (ES = 0.5).

In the first step of the analysis, Pearson correlation among boys revealed a relationship between relative lower limb length with COD time (r = − 0.45; *p* = 035) and FMI with COD (r = 0.56; *p* < 0.01) and AG (r = 0.52; *p* = 0.01). In females, only APHV correlated significantly with an index of reactivity (r = − 0.41; *p* = 0.04). No other significant correlation was found (Table 2).

**Table 2:** Linear regression models. *Statistically different values *p* < 0.05.

Variable		Sex	Regression model	Statistics			
Predictor	Dependent variable			x	X^2^	R^2^	*p*
FMI	COD	m	Linear	0.57	–	0.27	0.01*
			Polynomial	0.26	0.01	0.32	0.02*
RELATIVE LL	COD	m	Linear	− 0.45	–	0.19	0.04*
			Polynomial	0.27	0.04	0.33	0.01*
FMI	RA	m	Linear	0.53	–	0.39	0.01*
			Polynomial	0.16	0.03	0.28	0.04*
APHV	Index of reactivity	k	Linear	− 0.42	–	0.56	0.04*
			Polynomial	8.58	−0.39	0.25	0.04*

x—coefficient of the anthropometric measurement in regression function, x^2^—squared coefficients of the anthropometric measurement in regression function; R^2^—coefficient of determination; *p*-value—statistical significance.

**Figure 3:**
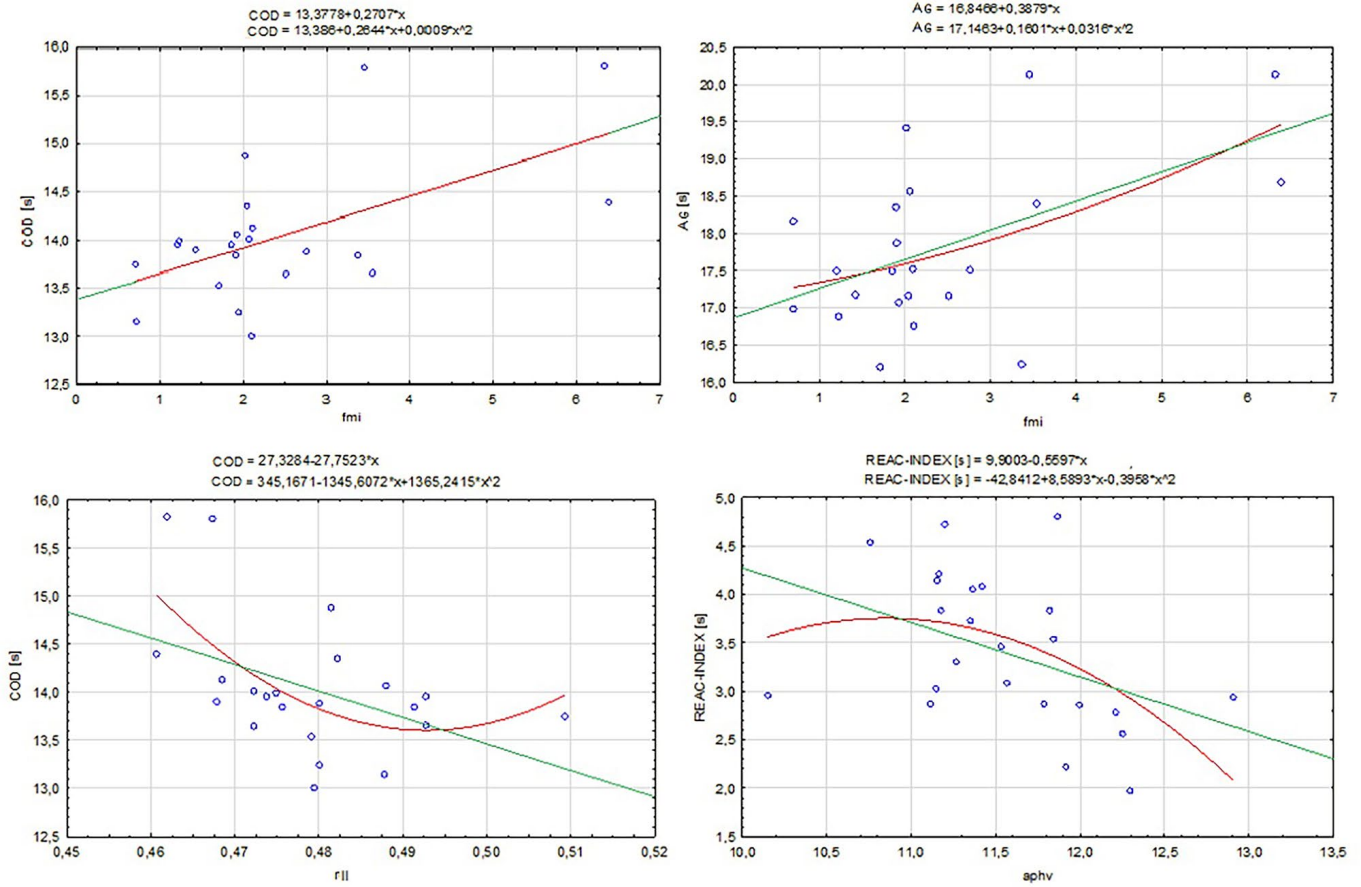
Linear and polynomial regression models.

To investigate the form of the relationship, a series of linear and polynomial regression models were constructed for correlated variables. Models in all approaches were statistically significant (*p* < 0.05) and revealed that analyzed variables highly explained COD-AG abilities in both sexes. In boys, FMI had a negative impact on the time of both motor tests showed in linear regressions (R^2^ = 0.27 for COD; R^2^ = 0.39 for AG), and polynomial regressions confirm linear form for COD-association FMI and model, which was similarly good fitted (R^2^ = 0.32) whereas for AG slightly curvilinear form (U-shaped) was worse fitted (R^2^ = 0.19). The positive impact was found for relative lower limb length (R^2^ = 0.19) in boys in the linear model. Thus, the polynomial regression showed that the curvilinear form fitted better in this association, suggesting that legs that are too short and too long decrease the COD-RA (R^2^ = 0.33). The optimal stature-leg length proportion is 0.50. The APHV was positively associated with the reactivity index in girls, and the model was well-fitted (R^2^ = 0.56). To deepen the exploration of association, the polynomial regression showed a curvilinear form (parabolic), but the model was worse fitted (R^2^ = 0.25) than linear. The peak value in this approach was 10.73. The regression models are presented in a scatterplot in Fig. 3.

**Figure 4:**
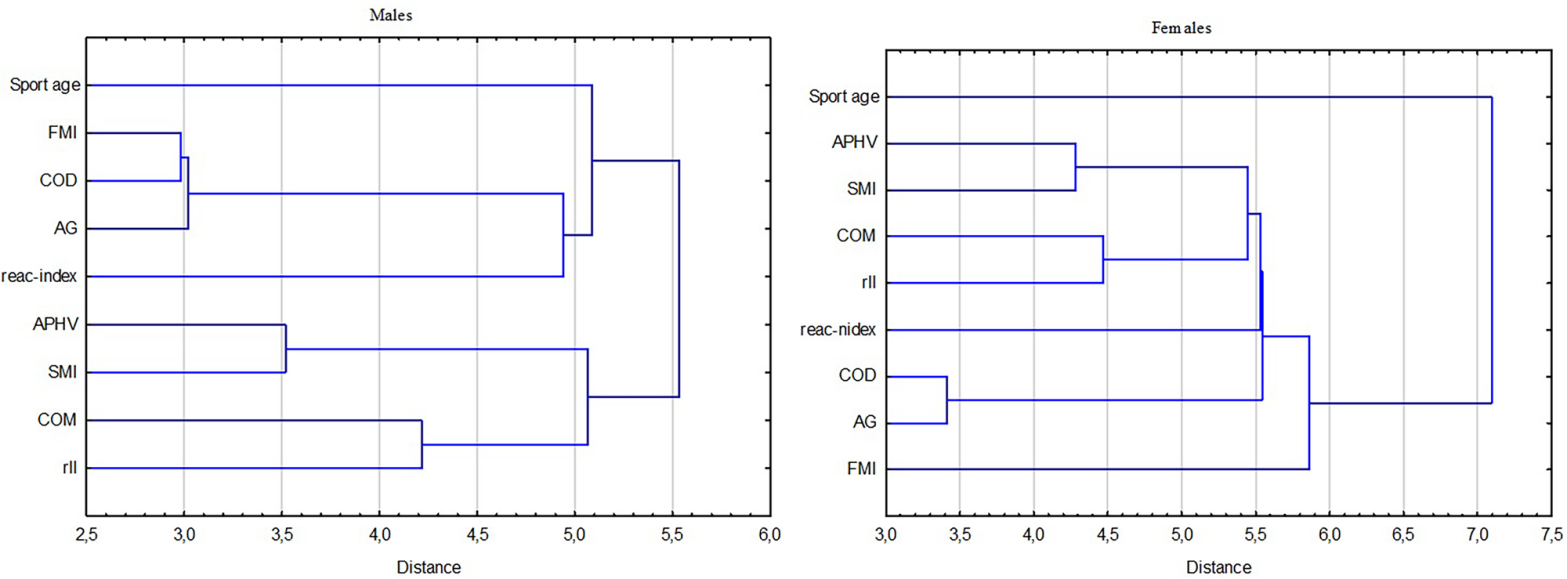
Dendrograms of cluster analysis present patterns in the relationship between maturity (APHV), body morphology, COD, and AG results (closely related measurements within a cluster are more similar, while measurements between clusters are more dissimilar).

The next stage of assessing the relationship between maturity, body morphology features, and motor skills was to study the patterns of the associations, investigating the clusters of variables concerning sex. Dendrograms illustrated the hierarchical structure of the analyzed variables based on the decreasing similarity of these traits (Fig. 4). Two clusters were revealed in the male group; the first contained COD and AG connected with FMI. It confirmed and strengthened the presented results of previous stages of analysis. This cluster was then attached to the sport age and index of reactivity. The second cluster contained closely connected APHV and SMI associated with COM and rLL. Results clearly showed the male-specific determination of the physical performance, which was related to the fat-to-height ratio and then anthropometric measurements, maturity, muscle-to-height ratio, or COM. In the female group, the relationship structure was different. COD-AG seemed more independent from the rest of the measurements, creating a small self-cluster. COD-AG was close to FMI, whereas sensorimotor ability was closer to the body morphology parameters cluster containing APHV. Sport age did not closely correspond with any variable.

## Discussion

This study aimed to explore COD and AG sex-specific relationship patterns with maturity and body morphology. Our results revealed a simple association between fat (FMI), leg length (rLL), and COD-AG skills in males, whereas APHV was associated with the index of reactivity. Analysis of the functional form of that relationship indicated that it is a linear relationship in the case of fat mass index. In contrast, in the case of COD- rLL, this association was a curvilinear form (U-shaped). The association between APHV and the index of reactivity takes a parabolic form. Clear sex-specific patterns were observed in clusters in males, with COD-RA closely associated with FMI. In contrast, in females, the patterns were more independent, loosely linked to the index of reactivity and morphological factors, and notably weakly correlated with both FMI and sports age.

Change of direction and agility are multifactor abilities that are essential in team sports^35^. Both are closely associated with speed, technique movement, acceleration-deceleration skills, and AG cognitive functions^35,36^. Many factors affected COD-AG skills^35^. In physical performance development among adolescents, a significant meaning is maturation, associated with rapid changes in body morphology features^37^. Despite not many studies considering relationship patterns of maturation and body dimension and morphology with COD and AG, this is an essential issue in the case of sport selection and skill development^38^. Identifying factors favorable or limited to COD and AG development states crucial information for coaches and players to define training forms and goals in every stage of long-term development^39^.

Our study showed that fat mass significantly negatively impacts COD-AG skills in boys, expressed in simple correlation and regression models. Also, cluster analysis confirm that FMI was close with COD-AG skills. An elevated fat percentage relative to body height corresponds to more surplus fatty tissue, resulting in heightened inertia. This necessitates increased force production per unit of lean mass to accomplish a specific change in velocity or direction^40^. A study by Esco et al.^41^ confirmed that young male soccer who presented elevated fat amounts were worse in motor performance tests. Despite fat, which, not surprisingly, had a deteriorative effect on the motor tests, those associations were not observed in girls. Illic et al.^42^ showed that studies on female volleyball players indicated that those players with lower body fat tend to be more agile. There is a need to remember that despite the possible association between fat and physical performance, it is not only one feature of body morphology that is affected by other factors that may also influence COD-AG^43^. Lastly, a systematic review with the meta-analysis by Matłosz et al.^44^ incorporates an analysis of body fat (BF) in competitive volleyball players. Significantly lower BF levels in male players compared to females are revealed, underscoring the necessity for the utilization of sex-specific calibration equations. Despite trends, the indication of performance across competitive levels by relative body fat did not correlate strongly with athletes’ performance levels.

In males, rLL plays a role in COD performance. However, the form of this relationship took a u-shaped form, which indicates that optimal length of lower limbs is required. The differences in leg length provide various run kinematics^45^. On one hand, shorter legs offer better maneuverability, whereas longer ones provide longer stride^46^. However, the balance between those characteristics seems optimal for better COD-AG.

Fat mass distribution and body dimensions proportion affected the center of mass placement^47^. Theoretically, it is essential to factor in the change of direction run in a pre-and non-planned manner^18^. However, our results did not reveal simple associations with this factor in both sexes. The explanation of this observation can be that COM in rest is not as important as an ability to lower COM during the pivotal action, and this parameter could be more associated with COD-AG skills; therefore, it is needed to analyze COM during testing and measure the COM changes what indicate future perspectives^18^. Moreover, anthropometric features state a linked factor and direct connections may not be enough to describe these relationship patterns. In boys, it was visible that the anthropometric factor states a single cluster, without FMI, which was closer to motor skills than others, which joined the COD-AG cluster as a single conglomerate. In girls, motor skills seem more independent from analyzed factors, and the cluster distances were higher than among boys. However, FMI also independently affected motor skills, and a cluster of anthropometric factors joined. This observation suggests a need to deepen the body morphology characteristic as a factor that influences COD-AG but in a multifactorial manner^48^.

In girls, there was no simple relationship in COD-AG that was also visible through the cluster analysis, which provides high variability. However, there was an observed parabolic relationship between APHV and sensorimotor ability, expressed as an index of reactivity, which states the difference between AG and COD times^8^. Generally, it is well known that more mature individuals are more developed morphologically and present higher physical fitness associated with muscle mass and nervous system development^49^. However, during maturation, some motor abilities could deteriorate in a timely manner because of the rapid growth of body dimensions, and, therefore, coordination disorders occur^50^. It was observed in the study by Gryko et al.^51^ that the training regimen for female basketball players aged 13–15 years should be tailored to meet their biological needs, particularly in areas such as jumping ability, endurance, and performance in the 20 m sprint test. Our study revealed that girls who start to maturate earlier tend to have a worse reactivity index. It seems surprising; however, there is a need to remember that the index of reactivity states a simple difference between not-planned and pre-planned tasks. The COD results indicated a specific physical potential in the absence of cognitive predispositions, the significance of which notably increases during unplanned tasks^52^. It is important to note that these findings do not imply that girls who mature earlier perform worse in COD-AG activities; instead, the observed difference is more pronounced, likely attributed to the uneven development of COD and AG skills. In other words, COD has improved more rapidly, while there is evident potential for enhancing AG through the development of cognitive functions. Considering the observed distinctions, the 16–17 chronological years is an appropriate time to introduce this element into agility training or even earlier.

This study was limited to only one age category. The extended analysis for another age category would provide deeper insight into COD-AG development. Moreover, dual-energy X-ray absorptiometry (DEXA) would be a more accurate method of determining biological age and body composition; thus, our methods used in this study are reliable^29^. We did not measure the center of mass during the motor tasks. There is a need to explore the COM shift during pivot tasks. Also, longitudinal studies are warranted to verify our observations. On the other hand, this study provides new insights into the knowledge about maturity and body morphology measures association with COD and AG skills in terms of sex-specific conditions in adolescent volleyball players. To date, there is a lack of studies considering the relationship between COD-AG skills and maturity and body morphology, and there is a lack of insight into the multifactor associations. In future research, there is a need to consider another age category, and measurements should be extended on COM analysis during the motor tasks during the lowering COM in shift, as well as other motor abilities in the multifactor approach^53,54^.

## Conclusion

In boys, two key morphological factors influence change of direction (COD) and agility (AG): optimal leg length relative to body height enhances maneuverability, while higher fat levels impair performance. Therefore, boys with lower body fat and optimal leg-to-body height ratios tend to perform better in COD-AG tasks. n girls, the relationship between COD-AG is complex and highly variable, as shown through cluster analysis. Our study also revealed a parabolic relationship between age at peak height velocity (APHV) and sensorimotor ability, indicating that earlier maturation might lead to a poorer index of reactivity. This suggests a need to account for the impact of maturation on unplanned tasks. Although earlier maturation does not always correlate with poorer COD-AG performance, differences may arise from uneven skill development in COD and AG. Cognitive functions, which can enhance AG, suggest that introducing agility training around age 16–17 or earlier could be beneficial. Thus, our results highlight the importance of selecting appropriate sports and maintaining suitable body composition to optimize performance in multidirectional tasks. Developing cognitive functions and tailoring training to accommodate non-changeable biological factors are also crucial. Sex differences in maturation and body morphology necessitate differentiated training approaches for male and female adolescent volleyball players to optimize COD-AG skills development.

## Data Availability

The data presented in this study are available upon request from the corresponding author.
